# Mechanism Assay of Honeysuckle for Heat-Clearing Based on Metabolites and Metabolomics

**DOI:** 10.3390/metabo12020121

**Published:** 2022-01-27

**Authors:** Hechen Wang, Lu Tian, Yiman Han, Xiaoyao Ma, Yuanyau Hou, Gang Bai

**Affiliations:** 1 State Key Laboratory of Medicinal Chemical Biology, College of Pharmacy, Nankai University, Tianjin 300353, China; wang210723@163.com (H.W.); chrissy0708@163.com (L.T.); hanyiman163@163.com (Y.H.); maxiaoyao@nankai.edu.cn (X.M.); 2 Tianjin Key Laboratory of Molecular Drug Research, Nankai University, Tianjin 300353, China

**Keywords:** metabolites, metabolomics, chlorogenic acid, swertiamarin, antipyretic, anti-inflammatory

## Abstract

Nonsteroidal anti-inflammatory drugs (NSAIDs), such as cyclooxygenase (Cox)-1/2 inhibitor, have emerged as potent antipyretics and analgesics. However, few herbs with Cox-1/2 inhibitory activity are commonly used for heat-clearing in China. Although these are known to have antipyretic activity, there is a lack of molecular data supporting their activity. Using the traditional Chinese medicine herb honeysuckle (Hon) as an example, we explored key antipyretic active compounds and their mechanisms of action by assessing their metabolites and metabolomics. Mitogen-activated protein kinase (MAPK) 3 and protein kinase B (AKT) 1 were suggested as key targets regulated primarily by chlorogenic acid (CA) and swertiamarin (SWE). CA and SWE synergistically inhibited the production of interleukin (IL)-1 and IL-6, alleviated generation of prostaglandin E2, and played an antipyretic role equivalent to honeysuckle extract at the same dose contents within 3 h. Collectively, these findings indicated that lipopolysaccharide-induced fever can be countered by CA with SWE synergistically, allowing the substitution of a crude extract of complex composition with active compounds. Our findings demonstrated that, unlike the traditional NSAIDs, the Hon extract showed a remote and indirect mechanism for alleviating fever that depended on the phosphatidylinositol-3-kinase–AKT and MAPK pathways by regulating the principal mediator of inflammation.

## 1. Introduction

Starting with aspirin, nonsteroidal anti-inflammatory drugs (NSAIDs), which target cyclooxygenase (COX)-1/2, have emerged as potent antipyretics and analgesics [1]. However, almost no heat-clearing herbal medicines containing COX-1/2 inhibitors based on traditional Chinese medicine are being used for alleviating fever. One of the few examples of plants that are being used is honeysuckle (Hon), which refers to the flower buds of *Lonicera japonica* Thunb., possessing as it does potential anti-pyretic and anti-inflammatory activities, and which is widely used clinically after extraction [2]. So far, more than 140 phytoconstituents have been identified from honeysuckle extracts. In addition to essential oils, an abundance of flavones, organic acids, iridoids, and triterpenoid saponins have also been found [3]. However, the key component responsible for its antipyretic activity and its corresponding target remain unknown. Therefore, it is important to investigate the complex mechanism behind antipyretic activity exerted by these active compounds, as this will provide the knowledge required to precisely tailor therapies for various conditions, such as exopathogenic diseases, sores, carbuncles, and some infectious diseases.

Natural products (NPs) remain the most prolific source of inspiration for the development of drugs [4]. The novel modes of action exhibited by NPs have always made them a subject of great interest in medicine [5]. With the exploration of NPs has become more common, increased access to biological and chemical data, and the development of data analysis algorithms, and integration of computational methods in NP drug discovery pipelines may be expected to increase significantly [6]. Data pre-processing using convolutional neural networks reportedly performed better in peak alignment and identification from liquid chromatography–mass spectrometry (LC–MS), compound identification and quantification, and integration and interpretation of multi-omics data [7]. Considering the complex and elusive nature of botanical products and their metabolism in the human body, metabolomic data generated for herbal drugs are large compared to those for single compounds. Combining information from different sources, that is, chemical, biochemical, biological, and in silico, with advanced computer algorithms and effectively handling big data can open new possibilities for NP research [8]. Unfortunately, the integration of multi-omics data—those for the structures/components and for their targets—remains a challenge.

Here, we used honeysuckle as a model herb and its extract to screen the key bioactive ingredients and potential protein targets in order to explore the antipyretic mechanism based on metabolites and metabolomic data. LC–MS coupled with molecular networking (MN) was adopted to analyze the prototype and metabolites in the blood and build a chemomic profiled network consisting of precursor ions. Virtual docking and annotation enrichment analyses were used to identify their structures and potential targets. Concurrently, a metabonomic assay was employed to detect the major reversal changes that were regulated by honeysuckle extractive preparation (HEP) administration in a lipopolysaccharide (LPS)-induced rat fever model. The interpretation of integrated data revealed chlorogenic acid (CA) and swertiamarin (SWE) as key compounds acting on both the phosphatidyl-inositol-3-kinase-protein kinase B (PI3K–AKT) and p38 mitogen-activated protein kinase (MAPK) pathways that are associated with fever.

## 2. Results

### 2.1. Global Metabolic Profiling Coupled with MN Identifies the Key Metabolites

**Figure 1:**
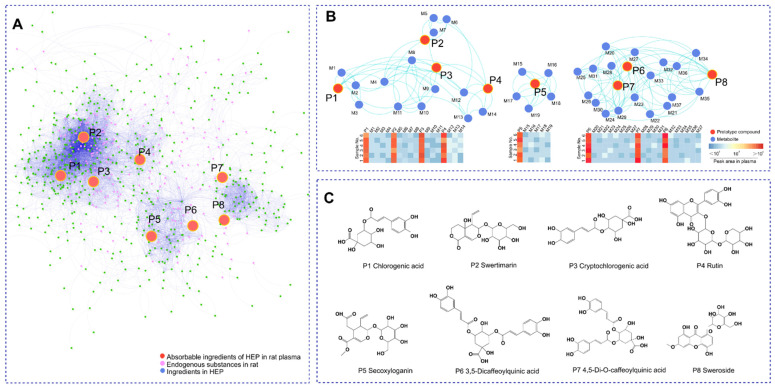
The identification of absorbable ingredients in HEP based on the MN assay. (**A**) Whole MN profiling of HEP, blank plasma sample, and administration plasma sample after oral HEP. (**B**) The network association diagram showed a clustering of the absorbable ingredients and their metabolites (**top panel**), as well as the relative contents of the absorbable ingredients and their metabolites. The minimum concentration was expressed as dark blue and the maximum concentration was expressed as dark red (**bottom panel**). (**C**) The structure of the key prototype compounds.

Global metabolite profiling based on negative and positive ion modes was performed to identify key metabolites in plasma derived from HEP, a honeysuckle oral liquid freeze-dried powder. The MS data of different groups were integrated into the Global Natural Product Social Molecular Networking (GNPS) platform. As shown in Figure 1A, the MN map contained 1371 precursor ions, including 297 clusters (node ≥ 2) and 313 single nodes. Interestingly, compared with the overlapped metabolite distribution in HEP- and HEP-treated plasma (Figure 1B), a total of eight main prototype components (P1-8) showed a significant difference in dysregulated metabolites. Based on the exact mass measurements and fragmentation patterns confirmed from references, as well as the Agilent natural product MN database, these compounds were identified as CA, SWE, cryptochlorogenic acid, rutin, secoxyloganin, 3,5-dicaffeoylquinic acid, 4,5-di-O-caffeoylquinic acid, and sweroside (detailed in Table 1), as shown in Figure 1C. The prototype-derived 37 metabolites (M1-37) linked by molecular weight profiling are listed in Table S1. The results showed that the potentially effective compounds were divided into three parts: polyphenol acids, flavonoids, and iridoids. Based on the *m/z* intensity response (Figure 1B, bottom panel), the eight potentially effective compounds were selected and used for the following target prediction by the PharmMapper website.

### 2.2. Integrated Analysis Reveals the Core Mechanism of Antipyretic Effects

**Figure 2:**
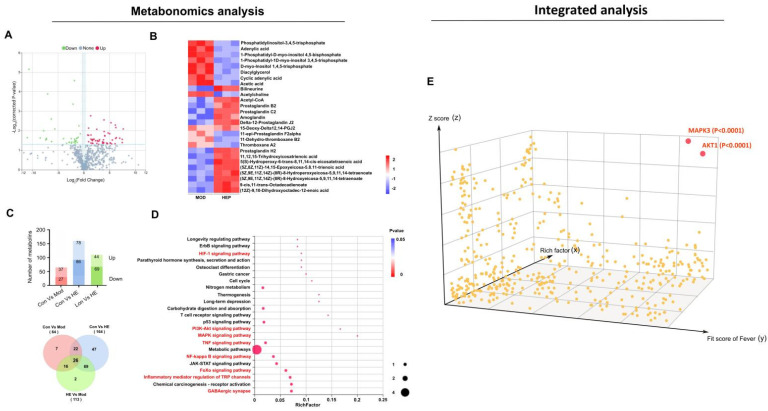
(**A**) Volcano plot of the quantitative metabolites in plasma after HEP administration according to fold change and corrected *p*-value. Metabolites with significant variation in abundance are shown as red (**up**) or blue (**down**) dots. (**B**) Heat map of cluster analysis illustrates the key related metabolites. (**C**) Metabonomic analysis of plasma. Statistical analysis of differential metabolites among experimental groups (**top panel**). Venn diagram analysis of the differential metabolites (**bottom panel**). (**D**) The 23 significantly enriched KEGG pathways. The x-axis shows rich factor; the y-axis corresponds to the KEGG pathway. The fever and inflammation pathways are shown in red. (**E**) The systematic integrative analysis of all the proteins associated with fever based on metabolites and metabolomics data. The x-axis represents the protein’s fit factor for fever from GeneCards; the y-axis represents the protein’s rich factor on the key pathway from KEGG; the z-axis represents the target protein’s docking score rich from PharmMapper. Adjusted *p*-values of different correlation scores were used to enable robust statistical interpretation.

To explore the underlying molecular mechanisms, the corresponding plasma samples were subjected to metabolomic analysis by LC–MS. As shown in Figure 2A, 289 different metabolites out of the total 952 were found in the quantitation to have a fold-change of ≥1.29 or ≤0.91 and corrected *p*-value ≤ 0.05. The detailed differential metabolites are shown in Figure 2B. According to the Venn diagram analysis, only 26 metabolites were identified in all the comparison groups (Figure 2C). A total of 23 pathways were annotated using KEGG pathway enrichment analysis (Figure 2D). Among these, eight key pathways namely, HIF-1, PI3K–AKT, MAPK, TNF, NF-kappa B, FoXo signaling pathways, inflammatory mediator regulation of TRP channels, and GABAergic synapse, were related to fever and inflammation.

To understand the key action nodes of HEP in fever, all associated proteins were used in the integrated analysis involved in the fever-pathway proteins (273) enriched from the above metabonomics, target proteins (400) were predicted from the key chemicals via PharmMapper, and fever-associated proteins (653) were obtained from GeneCards. A 3D diagram was constructed based on the correlation scores. As shown in Figure 2E, based on metabolites and metabolomics assays and system integration, only two distinct differential targets were explored: MAPK3 (ERK1) and AKT1, regulated by CA and SWE, respectively (detailed in the Supplementary Materials).

### 2.3. The Antipyretic Effects of CA Combined with SWE

**Figure 3:**
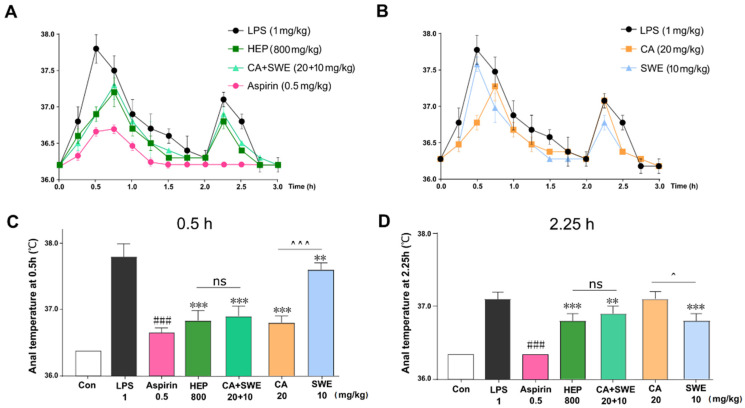
(**A**,**B**) Anal temperature curve of Aspirin, HEP, CA+SWE (**left panel**) and CA, SWE (**right panel**) in the first 3 h after intraperitoneal injection of LPS. Values are given as means ± standard deviation (SD) (*n* = 10). (**C**,**D**) Anal temperature at 0.5 h and 2.25 h for each group. ^###^ *p* < 0.001, Aspirin vs. LPS; ** *p* < 0.01, *** *p* < 0.001, drug intervention group vs. LPS; ^ *p* < 0.05, ^^^ *p* < 0.001, CA vs. SWE; ns, not significant (*n* = 10).

To verify whether the combination of CA and SWE played an alternative antipyretic role with HEP, the same dose of phenolic acids (CA 20 mg/kg) and iridoid glycosides (SWE 10 mg/kg) in HEP, which was replaced by Hon oral liquid, was used to evaluate the antipyretic effects in the LPS-induced rat fever model. As expected, LPS-induced fever can be significantly suppressed by aspirin. Similarly, the HEP and CA+SWE groups showed almost the same antipyretic effect within 3 h (Figure 3A, left panel). In the first half of the observation period, the effect of CA alone was better than that of SWE (Figure 3B, right panel). For a detailed comparison, the anal temperature of each group was compared at two peak times. Interestingly, there was no significant difference between the HEP and CA+SWE groups. Compared to the SWE group, CA had a better effect in the first 0.5 h (*p* < 0.001). The effect of SWE was better than that of CA at 2.25 h (*p* < 0.05) (Figure 3C,D).

### 2.4. The Potential Mechanism of CA and SWE against Fever

**Figure 4:**
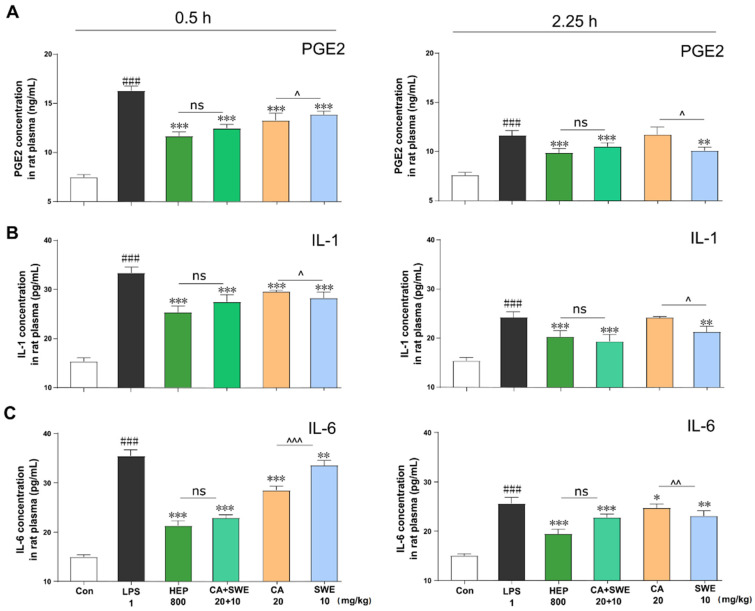
HEP, CA, and SWE downregulated the expression of pro-inflammatory cytokines in LPS-induced fever rats. The protein expression levels of PGE2 (**A**), IL-1 (**B**), and IL-6 (**C**) in the plasma at 0.5 h and 2.25 h were tested with ELISA kits. ^###^ *p* < 0.001, LPS vs. self-control; * *p* < 0.005,** *p* < 0.01, *** *p* < 0.001, drug intervention group vs. LPS; ^ *p* < 0.05, ^^ *p* < 0.01, ^^^ *p* < 0.001, CA vs. SWE; ns, not significant (*n* = 6).

To evaluate the effect of CA and SWE on the expression of downstream inflammatory factors by regulating MAPK3 and AKT1 targets, the expression of interleukin (IL)-1, IL-6, and prostaglandin E2 (PGE2) was also assessed. As shown in Figure 4A–C, compared to the LPS group, all groups showed significantly reduced levels of PGE2, IL-1, and IL-6. As expected, there was no significant difference between the HEP and CA+SWE groups (*p* > 0.05). Moreover, the result indicated that CA is better at downregulating IL-1 level than SWE, both at 0.5 and 2.25 h (*p* < 0.01). It was suggested to target MAPK3 (ERK1) signaling. Extracellular signal-regulated protein kinases 1 and 2 (ERK1/2) are signal transducers of IL-1β via the p38 MAPK pathway in inflammation processes [9]. SWE exhibited an inhibitory effect on IL-6 at 0.5 h (*p* < 0.001), which was consistent with previous findings indicating SWE inhibition of phosphorylation of AKT and alleviation of the PI3K–AKT signaling pathway [10]. Collectively, these findings suggest that the resultant LPS-induced fever can be rescued by the synergetic effect of CA with SWE and that the combined action of the p38–MAPK and PI3K–AKT pathways leads to a better antipyretic effect.

## 3. Discussion

Fever is an acute-phase complex physiological response by a host immune system against pathogens. The activation of peripheral immune cells by an infectious agent increases the generation of several inflammatory mediators. The first cytokine induced by immune challenge is TNF-α, followed by IL-1, IL-6, and other cytokines [11]. Systemic inflammation is often modeled by administering bacterial LPS, which secretes several agents, triggering autonomic and behavioral thermo-effector responses that cause either fever or hypothermia [12]. In mammals, PGE2 is the principal mediator of fever. IL-1 and IL-6 in the blood activate the expression of COX2 and PGS through their receptors on brain endothelial cells, evoking fever by eliciting PGE2 synthesis [13]. These PGE2 and pro-inflammatory cytokines induce an increase in body temperature.

As is widely known, the Toll-like receptor (TLR)-mediated PI3K–AKT and p38–MAPK pathways are involved in resisting pathogenic invasions. These pathways utilize transcription factors, such as NF-kB and AP-1, and further regulate the production of IL-1 and IL-6 [14]. Evidence suggests that the PI3K–Akt, MAPK, and NF-κB pathways participate in the inflammation process [15]. The extracellular signal-regulated kinases ERK1/2 and p38 play key roles in the MAPK pathway. The phosphorylation of ERK1/2 and p38 promotes the production of TNF-α, IL-6, and PGE2 [16]. Meanwhile, IL-1β induction of IL-6 production by activation of the p38 MAPK–NF-κB signaling pathway with a post-transcriptional mechanism [17], and the expression of IL-6, which is induced by IL-1β, was significantly reduced by p38–MAPK inhibition [18]. Hence, inhibiting the expression of IL-1 can simultaneously alleviate IL-1-triggered IL-6 expression and protein secretion [19].

In principle, the absorbed components in vivo, having sufficiently prolonged exposure may be directly associated with therapeutic efficacy [20]. Therefore, identifying the key bioactive components via global metabolic profiling, while focusing on the pathological effects, may be meaningful. In contrast to classic NSAIDs, few COX-1/2 inhibitors have been identified in commonly used clinical antipyretic herbs. Owing to the large number of compounds in the herbs and limited information about the huge regulatory network, accurately determining the most bioactive compound, its target, as well as the mechanism of action, remains challenging [21].

Here, MN based on approximate hierarchical clustering was used to find the main exposure prototype or metabolites in the blood from HEP. Chrom Align Net algorithm, and Mass-MetaSite were used to match and identify these components. The uppermost bioactive compound was utilized for target prediction using PharmMapper. Integrated biological data interpretation benefited from multi-omics data conversion and the disease-associated target database can improve the possibilities for target discovery [22]. Hence, the multi-dimensional protein targets, including the antipyretic-related targets from GeneCards, metabolomics-enriched targets from HEP intervention, and molecular docking targets were integrated. Through our integrated analysis processes, CA and SWE were found to be the key ingredients. It was known that CA attenuated different stimuli-induced ulcerative colitis or toxicity through the MAPK–ERK–JNK or MAPK–AKT pathways [23,24]. SWE was demonstrated to target the AKT PH domain, deactivated the phosphorylation of AKT, and presented significant anti-inflammatory activity [10]. Organic acids were indicated as the dominant compounds responsible for anti-inflammatory effects [25]. The above evidence also supported our results from another aspect.

In conclusion, our findings indicate that CA targets MAPK3, regulating the expression of both IL-1 and IL-6 and playing a vital role compared to SWE, which acts on the PI3K–AKT pathway to reduce the production of IL-6 via the AKT target. By attenuating these principal pro-inflammatory cytokines and their driving PGE2 expression, CA with SWE synergistically can simulate the effect of HEP complexes for relieving fever. This paper provides a feasible solution by using metabolite and metabolomics analysis to uncover the associations of complex systems.

## 4. Materials and Methods

### 4.1. Reagents and Chemicals

The sample of HEP directly replaced by honeysuckle (Jinyinhua) oral liquid (lot no. 635031, each package 20 mL, about 90 mg crude drug per ml) was produced by Zhenao Honeysuckle Pharmaceutical Co., Ltd. (Xianning, China). Chlorogenic acid (CA) and swertiamarin (SWE) (purity > 98%) were procured from Meilunbio (Dalian, China). Aspirin was obtained from Solarbio (Beijing, China). Rat IL-6, IL-1β, and PGE2 enzyme-linked immunosorbent assay kits were purchased from Thermo Fisher Scientific (Waltham, MA, USA) and LPS from Macklin (Shanghai, China). Moreover, LC–MS-grade methanol, acetonitrile, and formic acid (99.5+%) were purchased from Fisher Scientific, Inc. (Pittsburgh, PA, USA) and used for the preparation of mobile phases and solutions.

### 4.2. Animals

Experimental Sprague Dawley rats (8 weeks old, male, weighing 200 ± 10 g) were purchased from Beijing Vital River Laboratory Animal Technology Co., Ltd. (Beijing, China). All animals were fed a standard diet (Trophic Animal Feed High-tech Co. Ltd., Nantong, China) and given water ad libitum under controlled environmental conditions (temperature, 22 ± 2 °C; humidity, 45% ± 5%; light/dark cycle, 12 h/12 h).

After adaptation to the standard laboratory conditions for two weeks, the experimental rats were randomly allocated to the following groups (*n* = 10): control group (Con), Mod group (LPS, 1 mg/kg), Aspirin (0.5 mg/kg), HEP group (800 mg/kg crude drug), CA group (20 mg/kg), SWE group (10 mg/kg), and CA+SWE group (20 mg/kg CA plus 10 mg/kg SWE). In addition to the Con group, all groups were established by administering intraperitoneal injections at the same dose of LPS (1 mg/kg). After LPS injection, intragastric administration of Aspirin, HEP, CA, SWE, or CA+SWE was carried out immediately. The rectal temperature was measured with a rectal probe coupled to a digital thermometer (Taimeng, Sichuan, China) every 15 min for 3 h, and the temperature was calibrated for 15–30 s before readings were taken. All measurements were performed at a stable ambient temperature of 23 ± 1 °C.

### 4.3. Plasma Sample Preparation

First, plasma samples were obtained at different time points after oral administration of HEP (800 mg/kg). Blood samples were taken from retroorbital blood, and 100 µL of frozen plasma samples were thawed and placed into centrifuge tubes (1.5 mL). The samples were then thoroughly mixed with 200 µL of methanol and vortexed for 5 min. The solutions were centrifuged at 8000 rpm/min for 10 min. The supernatants at 0.5 h were filtered through a 0.22 µm membrane for metabonomic analyses. Similarly, a plasma mixture at 0.15, 0.5, 1.0, and 2.0 h was used for metabolite identification. Meanwhile, the plasma after administration was also collected at 0.5 h and 2.25 h and centrifuged at 8000 rpm/min for 10 min. The supernatants were then used for PGE2, IL-1, and IL-6 analyses by enzyme-linked immunosorbent assay.

### 4.4. UPLC–MS Analysis for Metabolites

An Align 1290 Infinity II UPLC combined with the 6550 iFunnel quadrupole time of a flight LC–MS system was used in the proofing and identification of metabolites (the conditions of chromatographic and MS–MS are described in the Supplementary Materials). Chrom Align Net software was used to identify the metabolites [26].

### 4.5. Data Processing

**Figure 5:**
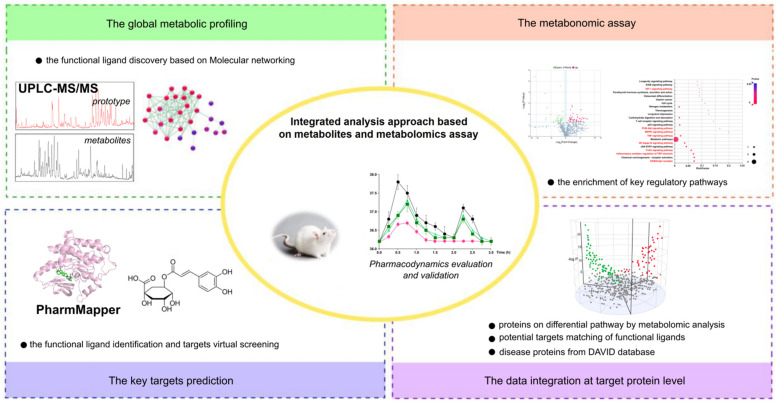
Workflow of the integrated analysis approach based on metabolites and metabolomics assay approach. The process including LC–MS combined MN screening to obtain the prototype compounds and its metabolites; the enrichment of the key target proteins from metabonomics assay; target prediction of potential bio-active compounds; and integrative analysis and validation of biological effect.

A comprehensive approach, including global metabolic profiling by MN based on MS–MS coupled with a series of integrated analysis, was used to discover and identify key compounds. The workflow of functional ligand discovery from metabolic profiling and the elucidation of the antipyretic mechanism via metabolomic data was performed, as shown in Figure 5.

For molecular network construction, the MS–MS data for metabolites were collected and converted to mzXML format using Proteo Wizard software (www.proteowizard.sourceforge.net, Proteo Wizard, Palo Alto, CA, USA) and uploaded separately to the GNPS platform (https://gnps.ucsd.edu, UCSD, San Diego, CA, USA) (accessed on 16 November 2021). The GNPS parameters were set as follows: mass error of less than 0.02 Da, matched peaks greater than 6, and cosine score greater than 0.50. Next, the merged molecular network was successfully obtained according to our reported method [27]. Finally, Cytoscape software v 3.7.1 (www.cytoscape.org, NRNB, Hill St, San Diego, CA, USA) (accessed on 16 November 2021) was used to build the molecular network.

For metabolomic analyses, the operations were performed according to our previous study [21]. A Q-Exactive HF X mass spectrometer (Thermo Fisher Scientific, Waltham, MA, USA) was used for metabolite detection. After pretreatment of the rat plasma by deproteinization, Compound Discoverer software (version 3.0; Thermo Fisher Scientific, USA) was used for further data analysis, and the BGI Library, mzCloud, and ChemSpider databases were used as the search databases.

To identify the key targets, the PharmMapper database (http://www.lilab-ecust.cn/pharmmapper/) (accessed on 16 November 2021) was used to predict key metabolites from HEP [27]. Python was adopted for data integration of all proteins, including differential pathways by metabolomic analysis, potential targets matching by the PharmMapper, disease proteins associated with fever, and the DAVID database. The intersect protein was visually displayed using PEAC seq (https://peac.hpc.qmul.ac.uk/) (accessed on 22 November 2021).

### 4.6. Statistical Analysis

Data are shown as means ± standard deviation. For single comparisons, significant differences between the means were determined by a one-way ANOVA Ordinary test. Statistical significance was set at *p* < 0.05. All data were processed using GraphPad Prism 7 software (GraphPad Software, La Jolla, CA, USA).

## Figures and Tables

**Table 1:** Related MS information of identified compound in HEP.

Name	t_R_ (min)	*m/z*	Formula	MS/MS *(m/z)*	Fit Score	Identification
P1	3.487	355.1027	C_16_H_18_O_9_	115.0277 91.6649	99.7	Chlorogenic acid
P2	16.354	375.1284	C_16_H_22_O_10_	121.6451 137.7673	99.8	Swertiamarin
P3	4.372	355.1027	C_16_H_18_O_9_	84.399 154.1601	99.7	Cryptochlorogenic acid
P4	14.265	611.1608	C_27_H_30_O_16_	63.9512 360.5813	99.8	Rutin
P5	10.242	405.1403	C_17_H_24_O_11_	68.8756 124.453	99.8	Secoxyloganin
P6	7.249	516.4517	C_25_H_24_O_12_	77.3744 248.0019	99.8	3,5-dicaffeoyl qunic acid
P7	8.225	355.1027	C_16_H_18_O_9_	82.6558 329.2093	99.7	4,5-Di-O-caffeoyl quinic acid
P8	12.347	359.1334	C_16_H_22_O_9_	62.7029 242.1572	99.8	Sweroside

## Data Availability

All data presented in this study are available from the authors upon request.

## Supplementary Materials

The following supporting information can be downloaded at: https://www.mdpi.com/article/10.3390/metabo12020121/s1, 1. Supplementary methods of Plasma sample preparation, UPLC-MS Analysis for metabolites, 2. Related information of the metabolites of HEP in MS data, 3. Data of intergrative analysis.
