# Supplementary material for: Mechanism Assay of Honeysuckle for Heat-Clearing Based on Metabolites and Metabolomics
Source: Metabolites. 2022 Jan 27;12(2):121. doi: 10.3390/metabo12020121 (PMC8874459; doi:10.3390/metabo12020121)
Supplement: Supplementary file 1 [file metabolites-12-00121-s001.zip › metabolites-1508491-supplementary.pdf]

Supplemental Information for

Original article

Mechanism assay of Honeysuckle for heat-clearing based on metabolites and metabolomics

Supporting Information Available:

1. Supplementary methods of HEP and Plasma sample preparation, UPLC-MS Analysis for metabolites
2. Related information of the metabolites of HEP in MS data
3. Data of intergrative analysis

1. Supplementary methods of Plasma sample preparation, UPLC-MS Analysis for metabolites

The sample of honeysuckle extractive preparation (HEP) directly replaced by Honeysuckle oral liquid (lot no. 635031, Each package 20 ml, about 90 mg crude drug per ml), which was produced by Zhenao Honeysuckle Pharmaceutical Co., Ltd. (Xianning, China). HEP samples were prepared by freeze-drying to the dry extract, then mix the extract with methanol in 1mg/ml. Plasma samples were obtained at different time points (15, 30, 60, and 120 min) after oral administration of HEP (800 mg/kg). Then, HEP and mixed plasma were identified by Q-TOF-MS.

UPLC analysis was performed on the 1290 Infinity II UPLC system equipped with a binary solvent system and an automatic sample manager (Agilent™ Corporation). The Agilent InfinityLab Poroshell 120 EC-C18 (2.1 mm × 50 mm, 1.9 μm) maintained at 30 ºC was used for separation. The mobile phase consisted of water (A) (including 0.1 % formic acid, v/v) and methanol (B), and the flow rate was set at 0.4 mL/min. Gradient elution was carried out as shown below, 5% B at 0 min, 5-50% B at 0-15 min, 50-100% B at 15-30 min. For analysis, 2.0 μL was injected into the above-described UPLC system.

The UPLC system was used in combination with the 6550 iFunnel Quadrupole Time of Flight LC/MS, and the mass spectra were collected using the electrospray ionization source (ESI) in the full-scan mode from m/z 50 to 1200 at the drying gas (N_2) flow rate of 9 L/min and the temperature of 350℃ in positive mode, respectively. Moreover, the conditions adopted for ESI source were optimized as follows: nebulizer pressure of 45 psig, capillary voltage of 4000 V, fragment voltage of 125 V, and skimmer voltage of 65 V. The MS spectra were collected at 1.4 spectra/s. To guarantee veracity and reproducibility, Lucille-enkephalin was utilized as the lock mass (m/z=121.050873, 149.02332, 922.009798).

Thereafter, the MS/MS data were collected and converted to mzXML format by Proteo Wizard software (www.proteowizard.sourceforge.net), they were uploaded separately to the GNPS platform (https://gnps.ucsd.edu). The GNPS parameters were set as follows: mass error of less than 0.02 Da, matched peaks greater than 6, and cosine score greater than 0.50. Finally, Cytoscape software v3.7.1 (www.cytoscape.org) was used to build the molecular network.

2. Related information of the metabolites of HEP in MS data

The TIC chromatograms of HEP

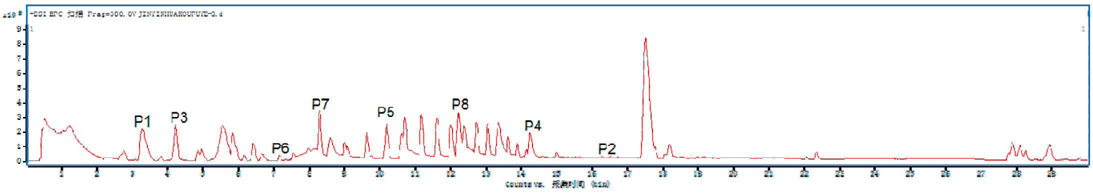

Figure S1 TIC chromatograms of HEP

Table S1. Related MS information of identified compound in HEP

Name	RT (min)	m/z	Formula	MS/MS (m/z)	Fit score	Identification
P1	3.487	355.1027	$C_{16}H_{18}O_9$	115.0277<br>91.6649	99.7	Chlorogenic acid
P2	16.354	375.1284	$C_{16}H_{22}O_{10}$	121.6451<br>137.7673	99.8	Swertimarin
P3	4.372	355.1027	$C_{16}H_{18}O_9$	84.399<br>154.1601	99.7	Cryptochlorogenic acid
P4	14.265	611.1608	$C_{27}H_{30}O_{16}$	63.9512<br>360.5813	99.8	Rutin
P5	10.242	405.1403	$C_{17}H_{24}O_{11}$	68.8756<br>124.453	99.8	Secoxyloganin
P6	7.249	516.4517	$C_{25}H_{24}O_{12}$	77.3744<br>248.0019	99.8	3,5-Dicaffeoyl qunic acid
P7	8.225	355.1027	$C_{16}H_{18}O_9$	82.6558<br>329.2093	99.7	4,5-Di-O-caffeoyl quinic acid
P8	12.347	359.1334	$C_{16}H_{22}O_9$	62.7029<br>242.1572	99.8	Sweroside

Table S2. Related MS information of identified compound in HEP

Name	RT (min)	*m/z*	Formula	MS/MS (*m/z*)	Fit score	Identification
M1	3.994	191.0491	$C_9H_8O_4$	87.7522<br>144.6726	88.7	Caffeic acid
M2	25.41	193.0704	$C_7H_{12}O_6$	75.3082<br>100.4683	91.1	Hexahydro-1,3,4,5-tetrahydr oxybenzoic acid
M3	25.25	165.0542	$C_9H_8O_3$	130.2439<br>96.9847	88.6	p-Coumaric acid
M4	4.835	183.0657	$C_9H_{10}O_4$	147.3945<br>71.6291	90.0	Dihydro caffeic acid
M5	25.211	175.0608	$C_7H_{10}O_5$	74.6901<br>121.6834	91.8	Shikimic acid
M6	3.362	180.0654	$C_9H_9NO_3$	124.5861<br>164.1134	89.6	Hippuric acid
M7	12.524	195.0425	$C_{10}H_{10}O_4$	128.122<br>136.0494	89.4	3-Hydroxy-4-methoxycinna mic acid
M8	26.556	195.0425	$C_{10}H_{10}O_4$	80.5806<br>115.3876	89.5	Ferulic acid
M9	11.322	171.0245	$C_7H_6O_5$	118.5762	91.7	Gallic acid

				92.7784		
M10	7.641	139.0397	$C_7H_6O_3$	113.4761<br>65.2376	88.0	p-Hydroxybenzoic acid
M11	2.507	199.0607	$C_9H_{10}O_5$	73.5804<br>153.9328	88.3	Syringic acid
M12	19.773	169.0472	$C_8H_8O_4$	67.4148<br>153.6583	88.5	Vanillic acid
M13	20.547	123.0447	$C_7H_6O_2$	55.4354<br>83.1203	91.4	Benzoic acid
M14	2.024	183.0656	$C_9H_{10}O_4$	141.1366<br>97.8113	90.6	Methyl Vanillate
M15	8.072	479.0830	$C_{21}H_{18}O_{13}$	107.8971<br>325.5979	89.7	Quercetin<br>3-O-β-D-Glucuronide
M16	18.801	478.0741	$C_{21}H_{17}O_{13}$	111.5251<br>141.0793	90.8	Quercetin glucuronic acid
M17	6.902	492.0871	$C_{22}H_{19}O_{13}$	106.0237<br>132.6761	90.7	Methylquercetin<br>glucurononic acid
M18	13.101	302.0417	$C_{15}H_9O_7$	150.6876<br>87.0066	91.3	Quercetin
M19	19.115	306.0722	$C_{15}H_{13}O_7$	66.4996<br>134.7178	91.2	Methyl isoquercetin
M20	3.814	377.1445	$C_{16}H_{24}O_{10}$	52.5618<br>127.0882	89.0	Loganic acid
M21	24.385	419.1542	$C_{18}H_{26}O_{11}$	72.3185<br>312.0128	89.1	Secoxyloganin methyl ester
M22	21.5	177.0542	$C_{10}H_8O_3$	109.8876<br>148.5308	90.5	Erythrocentaurin
M23	11.287	176.0704	$C_{10}H_9NO_2$	162.8376<br>150.6240	88.9	Gentianine
M24	2.774	611.1609	$C_{27}H_{30}O_{16}$	93.9951<br>334.8424	88.4	Kaempferol 3-gentiobioside
M25	17.575	419.1724	$C_{22}H_{26}O_8$	88.1328<br>229.2716	91.9	Syringaresinol
M26	2.181	347.1337	$C_{15}H_{22}O_9$	65.3085<br>139.9665	89.8	Aucubin
M27	12.394	513.1621	$C_{23}H_{28}O_{13}$	64.0394<br>195.7860	89.3	Picroside II
M28	10.583	495.1859	$C_{24}H_{30}O_{11}$	103.0549<br>70.6587	88.1	Harpagoside
M29	6.187	365.1443	$C_{15}H_{24}O_{10}$	130.9049<br>113.1133	90.3	Harpagide
M30	25.6	333.1542	$C_{15}H_{24}O_8$	129.3621<br>92.1502	91.6	Ningpogoside A
M31	3.32	489.1621	$C_{21}H_{28}O_{13}$	87.4778<br>116.6583	90.9	Cistanoside F
M32	3.764	639.2264	$C_{30}H_{38}O_{15}$	122.6474<br>74.3280	91.0	Cistanoside C
M33	3.459	149.0594	$C_9H_8O_2$	45.8770<br>99.9472	90.2	Cinnamic acid
M34	25.831	347.1335	$C_{15}H_{22}O_9$	72.3702<br>116.8159	90.4	Catalpol
M35	8.448	319.1752	$C_{15}H_{26}O_7$	69.2475<br>122.9523	91.5	Harpagide
M36	19.515	347.1335	$C_{15}H_{22}O_9$	94.9770<br>212.7348	92.0	Iridoid skeleton
M37	22.866	203.0917	$C_9H_{14}O_5$	96.0727<br>67.3242	89.9	Iridoid isomer

3. Data of integrated analysis

Intergrative analysis.xlsx
